# Eliciting Preferences for Health Insurance in Iran Using Discrete Choice Experiment Analysis

**DOI:** 10.15171/ijhpm.2019.29

**Published:** 2019-06-09

**Authors:** Ali Kazemi Karyani, Ali Akbari Sari, Abraha Woldemichael

**Affiliations:** ^1^ Research Center for Environmental Determinants of Health, Health Institute, Kermanshah University of Medical Sciences, Kermanshah, Iran.; ^2^ Department of Health Economics and Management, School of Public Health, Tehran University of Medical Sciences, Tehran, Iran.; ^3^ School of Public Health, College of Health Sciences, Mekelle University, Tigray, Ethiopia.

**Keywords:** Preferences, Health Insurance, Attributes, Discrete Choice, Iran

## Abstract

**Background:** The preferences of Iranians concerning the attributes of health insurance benefit packages are not well studied. This study aimed to elicit health insurance preferences among insured people in Iran during 2016.

**Methods:** A mixed methods study using a discrete choice experiment (DCE) approach was conducted to elicit health insurance preferences on a total sample of 600 insured Iranians residing in Tehran. The final design of the DCE included 8 health insurance attributes. Data were analyzed using conditional logistic regression models.

**Results:** The final model of this DCE study included 8 attributes, and the findings indicated statistically significant (*P*<.001) increase in the odds ratio (OR) of choosing health insurance at all levels of cost coverage except for the rehabilitation and para-clinical benefits, where at 70% cost coverage there was insignificant (*P*=.485) disutility (OR=0.95). With the increase in cost coverage level, the probability of choosing health insurance was significantly (*P*<.001) the highest for the private hospitals’ benefits (OR=2.82) followed by public hospitals’ benefits (OR=2.02) and outpatient benefits (OR=1.75), and the premium revealed statistically significant (*P*<.001) disutility (OR=0.96).

**Conclusion:** Our findings revealed that participants would be willing to choose health insurance plans with higher cost coverage of healthcare services and with lower premiums. However, the demographic characteristics, income, and health status of the insured individuals affected their health insurance preferences. The findings can contribute to the design of better health insurance policies, improve the participation of individuals in health insurance, and increase the insured individuals’ utility from the insurance benefits packages.

## Introduction

Health insurance is one of the financing mechanisms in health. Most countries use different ways of health insurance to reduce uncertainty in health expenditures by collecting predictable premiums from members.^1^ Health insurance has a considerably high positive effect on social protection and economic development, especially to vulnerable people such as the poor and rural residents.^2^

Article 29 of the constitution of the Islamic Republic of Iran states that all Iranians should have health insurance coverage. Public participation and revenues collected from the public are among the vital sources of financing the healthcare. Other main financing sources included health insurance payments and out of pocket payments. Generally, the Iranian health system is dominantly an insurance-based system.^3^

There is a mix of public and private health insurance schemes in the Iranian health system. However, the Social Security Insurance Organization (SSIO), and the Health Insurance Organization (HIO) formerly called the Medical Insurance Organization are the main ones. The Armed Forces Medical Services Insurance Organization, and the Imdad Committee Health Insurance are also other schemes that cover health insurance for the military and the poor, respectively.^3^ In 2013, the SSIO insured about 39 million people. This figure has increased to more than 42 million people in 2017, out of which about 8 million of them resided in Tehran province.^4^ Again, the HIO has insured another more than 32 million people in 2018.^5^

The healthcare financing challenges and the poor design of the insurance schemes are among the main issues that constrained the successful financial protection and service delivery in Iran. The insurance schemes that are designed based on diseases by ignoring the health, pricing, definition of tariffs, and benefits packages are not evidence-based.^6^ The designing of health insurance schemes should consider peoples’ preferences. The discrete choice experiment (DCE) method is a suitable method to assess peoples’ health insurance preferences. This method, for example, has been used to generate evidence concerning the Dutch peoples’ preferences among the different health insurance plans,^7^ and to elicit the health insurance preferences of different groups of people in Thailand.^8,9^

The demand for health insurance largely depends on its ability to meet the consumer’s needs, expectations and preferences.^9,10^ To the best of our knowledge, little evidence is available concerning the health insurance preferences of the Iranians. This study aimed to elicit the health insurance preferences of Iranians residing in Tehran using the DCE method. The findings are of paramount importance inputs for health insurance policy making and to provide people with health insurance benefits packages that incorporate their preferences in the context of Iran and perhaps beyond.

## Methods

### Study Area

Iran is one of the middle-income countries with a total population of about 80 million people for the year 2016 and spent about 6.9% of its gross domestic product (GDP) on health in 2014.^11^ The health system has both the private and public sectors. The public sector is responsible for the provision of primary, secondary and tertiary healthcare services, while the private sector provides secondary and tertiary services.^12^ Most of the secondary and tertiary healthcare services are under the coverage of health insurance. However, the services users have to pay coinsurance payments at the point of service delivery. These payments are higher in the private sector because the tariff is higher in the private sector than in the public one. The individuals with no health insurance coverage should pay all the charges, especially when they obtain the services from the private sector.^13^

About three-fourth (74.4%) of the Iranians are urban residents.^4^ In 2010, around 16% of the urban population did not have any health insurance coverage.^14^ In April 2014, the health sector evolution plan of Iran introduced a health insurance scheme to remove the financial barriers of access to healthcare services. This plan provided health insurance coverage for people who have no health insurance coverage by the HIO through the government’s financial support. For those who cannot afford the cost of health insurance, the premium was free. This intervention increased the coverage of health insurance in the country.^15^ The compulsory health insurance is the means to cover the rural residents and almost do not pay any premium.^16^ This study focused on the insured Iranians residing in Tehran, the capital of Iran, during 2016.

### Experimental Design

We applied a mixed methods study using a DCE to elicit health insurance preferences among the insured Iranians living in Tehran. This study involved 4 stages of the DCE method.^17-19^ Initially, we determined the attributes and attribute-levels from a list obtained by narrative reviews and interviews of nine experts. Reviewing literature, and interviewing of groups such as experts, and study population or a mix of these methods is a regular practice to determining the attributes in DCE studies. Thus, we performed review of literature and experts’ interview to extract the preliminary list of health insurance attributes. Then, 36 experts assessed the list and identified 8 eligible ones. The details of the attributes and attributes-levels are available elsewhere.^20^ The D-efficiency criteria have been used to design the choice set and select the most efficient attributes to be included in the final experimental design.^19^ The final design contained 24 choice set divided into 3 blocks. Each block had 8 choice set which again consisted of plans A and B. The experimental design of the choice set was performed using the SAS version 9.1.

We designed a questionnaire that consisted of 3 sections: the description of the research and informed consent, the socio-economic and demographic characteristics of the participants, and 3 blocks of 8 choice set. Therefore, we developed 3 versions of the questionnaire that were different only in the choice set (blocks). The participants were explained about the included attributes before proceeding the tasks, and the dominant choice set was used to test for consistency of the questions and to warm up the participants. In this phase, we excluded the participants who missed the correct answer to the dominant choice set. We used the experts’ opinion to validate and piloted the questionnaire. Each block of the choice set was pilot tested on 15 insured participants from the study area to ensuring the reliability and internal consistency of the tool. The pilot data were excluded from the analysis. Two trained data collectors obtained the data for the final analysis using an interview method. The research team has performed regular supervision to ensure the quality of the collected data. The attributes and attribute-levels of the health insurance in Iran and the choice set included in the analysis of our study are shown in Table 1 and Table 2, respectively.

**Table 1:** Attributes and Attribute-Levels of the Health Insurance in Iran

**Attributes**	**Levels**
Public hospitals benefits	Coverage of 60/90% of costs
Private hospitals benefits	Coverage of 50/70/90% of costs
Outpatient benefits	Coverage of 50/70/90% of costs
Dental care coverage benefits	Coverage of 40/70% of costs
Rehabilitation and para-clinical benefits	Coverage of 50/70/90% of costs
Long-term care benefits	Coverage of 50/70/90% of costs
Medical devices benefits	Coverage of 60/90% of costs
Premium	250 000/350 000/450 000 Rials

**Table 2:** One of the Choice Set Included in the Study

**Attributes**	**Plan A**	**Plan B**
Public hospitals benefits (cost coverage)	60%	90%
Private hospitals benefits (cost coverage)	90%	50%
Outpatient benefits (cost coverage)	70%	90%
Dental coverage (cost coverage)	40%	40%
Rehabilitation and para-clinical benefits (cost coverage)	70%	70%
Long-term care (cost coverage)	50%	70%
Benefits for medical devices (cost coverage)	60%	90%
Premium (IRRs)	450 000	350 000
Which of the health insurance plans would you like to choose (Please tick one box only)?	???Plan A o	Plan B o

### Sample Size

Determining the sample size of the DCE study is complex. Some suggest at least 30 participants for each subgroup such as age, gender, etc.^17^ Others recommend a threshold of 1000,^16^ and yet other researchers suggested a minimum of 500 to ensure the precision of the findings. Our study considered a threshold of 1000 and the sample size was calculated using the following formula^21^:

(1) $$S=\frac{nta}{c}$$

Where *S* represents the sample size, *n* is the total number of respondents, *t* is the total number of choice sets in the questionnaire, *a* is the number of alternatives in the choice sets, and *c* is the highest number of levels in the final attributes. The sample size in this study was 600 participants.

### Sampling Technique

Initially, we grouped the 22 municipalities in Tehran into 5 homogeneous clusters. Then, we selected one municipality from each cluster and a branch each from the SSIO and the HIO operating in that municipality randomly. Finally, we applied the proportionate allocation to population size technique to determine the sample size needed from each agency and selected the insured individuals using a systematic random sampling technique.

### Data Analysis

The random utility theory was the basis for the analysis of the DCEs data. Accordingly, consider that a person should choose between 2 alternatives A or B, and chose A. This indicates that alternative A provides more utility to the person than alternative B, and can be mathematically expressed as follows.^22^

*U (A, C) > U (B, C) * (2)

Where U is the utility derived from each alternative A and B, and C is a personal attribute which becomes effective upon choosing the alternative. As C is a common element both sides of equation 2, alternatively equation 2 can be expressed as follow:

*V (A-B) = U (A, C) - U (B, C) * (3)

Where V is indirect utility obtained from the alternative A compared with alternative B. The fitted utility function is expressed using a linear equation as follows:

*V=β*_1_ *Pubh + β*_2_ *Prih + β*_3_ *Outp + β*_4_ *Dent + β*_5_ *Rep + β*_6_ *Lt+ β*_7_ *Md + β*_8_ *Prem + ɛ * (4)

Where β_1_ to β_8_ are the coefficients of the benefit packages, and the attributes: Pubh (public hospitals benefits), Prich (private hospitals benefits), Outp (Outpatient benefits), Dent (dental coverage), Rep (rehabilitation and Para-clinical benefits), Lt (long-term care), Md (benefits for medical devices), and Prem (Premium) included in the analysis, and Ɛ is the error term. Assuming that the error terms have Logistic distribution, we applied the conditional logit regression model to analyze the data.^23^ This model assumes that the choices made are independent of irrelevant alternatives (IIA) which may be restrictive. Meanwhile, it is more useful to estimate the utility obtained from an attribute than using the more complicated models such as the nested logit and mixed logit models.^24^ The positive (negative) β coefficient indicates an individual’s utility (disutility) from the use of the chosen attribute. The McFadden R^2^ and χ^2^ tests were used to examine the goodness of fit of the models. We used the statistical software packages SAS 9.1 and Stata 13 (StataCorp, College Station, TX, USA) to perform the analysis.

## Results

### Attributes and Levels

The benefits from the public hospital services, private hospital services, outpatient services, dental care coverage, rehabilitation and para-clinical services, long-term care, medical devices (eg, Ortez, Protez, etc), and the health insurance premium were the attributes included in the final design. The service cost coverage levels for the private hospital benefits, outpatient benefits, rehabilitation and para-clinical therapy benefits, and long-term care benefits were 50%, 70%, and 90%, respectively. The base and service cost coverage

levels for the dental care were 40% and 70%, while for the public hospital benefits and the medical devices benefits the base and levels were 60% and 90%, respectively. The premium levels were 250 000, 350 000, and 450 000 Iranian Rials (IRRs), respectively. Each US dollar was equal to 32000 IRRs in the time of the study. The public hospitals were affiliated to medical universities under the supervision of the Ministry of Health and Medical Education, while the private hospitals provide services and owned by private health sector.

### Descriptive Statistics of Participants

Out of the total 600 study participants, males accounted for 327 (54.5%) (Table 3). The mean age was 41.48 ± SD = 14.67 years. Those participants with above 12 years of schooling accounted for 45.17%. Furthermore, 36.16% of the male and 16.50% of the female participants were employed, while 15.83% of the overall participants were retired. Again, 76% and 28% of the total study participants were male and female household heads, respectively. The monthly income for 41% of the households was less than twenty million IRRs, while for 31.5% it was 21 to 40 million IRRs. About 75% of participants had social security insurance, and 56.33% of participants had one or more chronic diseases.

**Table 3:** Descriptive Statistics of Insured Study Participants in Tehran, Iran; 2016

**Variables**		**Male**	**Female**	**Total**
		No. (%^a^)	No. (%^a^)	No. (%^a^)
Age	41 and younger	175 (29.17)	160 (26.67)	335 (55.83)
	Older than 41	152 (25.33)	113 (18.83)	265 (44.17)
Education level (years of schooling)	Low educated (≤ 12)	188 (31.33)	141 (23.50)	329 (54.83)
	High educated (>12)	139 (23.17)	132 (22.00)	271 (45.17)
Head of household	Yes	248 (41.33)	52 (8.67)	300 (50.00)
	No	79 (13.17)	221 (36.83)	300 (50.00)
Household income (in million IRRs)	≤ 20	156 (26.00)	150 (25.00)	306 (51.00)
	21-40	110 (18.33)	79 (13.17)	189 (31.50)
	>40	61 (10.17)	44 (7.33)	105 (17.50)
Type of basic health insurance	SSIO	249 (41.50)	206 (34.33)	455 (75.83)
	Others (HIO, etc)	78 (13.00)	67 (11.17)	145 (24.17)
Chronic condition (s)	Yes	175 (29.17)	163 (27.17)	338 (56.33)
	No	152 (25.33)	110 (18.33)	262 (43.67)
Overall total		327 (54.50)	273 (45.50)	600 (100.00)

Abbreviations: SSIO, social security insurance organization; HIO, health insurance organization. ^a^ Percentage for all the cells was calculated from total participants (dominator equals to 600).

### Preferences About Health Insurance

The regression analysis revealed statistically significant (*P* ≤ .001) associations between increasing the cost of coverage levels and the probability of choosing the health insurance for all the attributes except for the 70% coverage level for the rehabilitation and para-clinical benefit which was insignificant (*P* = .485). Increasing the cost coverage level from 50% to 90% significantly (*P* < .001) increased the odds of choosing the health insurance for the private hospital benefits more than twice (odds ratio [OR] = 2.82). Increasing the cost coverage level for the public hospital benefits from 60% to 90% also increased the probability of choosing health insurance for the public hospital benefits by about twice (Table 4).

**Table 4:** Regression Model Eliciting Preferences of Attributes of Health Insurance

**Attributes**	**Cost Coverage**	**β**	**SE**	***P*** **Value**	**OR**
Public hospitals benefits (base: 60%)	90%	0.705	0.036	<.001	2.023
Private hospitals benefits (base: 50%)	70%	0.585	0.053	<.001	1.795
	90%	1.040	0.055	<.001	2.824
Outpatient benefits (base: 50%)	70%	0.270	0.050	<.001	1.310
	90%	0.561	0.052	<.001	1.753
Dental coverage (base: 40%)	70%	0.451	0.078	<.001	1.570
Rehabilitation and para-clinical benefits (base: 50%)	70%	-0.047	0.067	.485	0.954
	90%	0.484	0.058	<.001	1.622
Long-term care benefits (base: 50%)	70%	0.259	0.074	<.001	1.306
	90%	0.356	0.076	<.001	1.428
Medical devices benefits (base: 60%)	90%	0.209	0.057	<.001	1.232
Premium (per 10 000 IRRs)	-	-0.036	0.004	<.001	0.965
Observation	9600				
McFadden's R^2^	0.19				
Log likelihood	-2695				
LR chi2(1^2^)	1263				
Prob > chi2	<0.001				

Abbreviations: SE, standard error; OR, odds ratio.

### Subgroup Analysis

*Preferences by Gender:* The findings revealed that both males and females had higher utilities from the inpatient and outpatient benefits than from the other attributes. At 70% cost coverage for the rehabilitation and para-clinical benefits, females had disutility (OR = 0.86), and males had some utility (OR = 1.04). The public hospitals’ benefits at 90% cost coverage provided relatively a higher utility for males than for females (OR: 2.23 vs. 1.81), while females had considerably higher utility from the private hospitals’ benefits than males (OR: 3.15 vs. 2.58). Both males and females showed an increase in the utility from the outpatient benefits with an increase in cost coverage (Table 5).

**Table 5:** Health Insurance Attributes Preferences of Participants by Gender, Age, and Educational Level in Tehran, Iran; 2016

**Attributes**	**Cost Coverage**	**Gender**				**Age Group (y)**				**Educational Level**			
		**Male**		**Female**		**41 And Younger**		**Older Than 41**		**Lower Educated**		**Higher Educated**	
		**β (SE)**	**OR**	**β (SE)**	**OR**	**β (SE)**	**OR**	**β (SE)**	**OR**	**β (SE)**	**OR**	**β (SE)**	**OR**
Public hospitals benefits (base: 60%)	90%	0.801 (0.049)^a^	2.228	0.594 (0.053)^a^	1.811	0.683 (0.048)^a^	1.980	0.742 (0.054)^a^	2.100	0.768 (0.049)^a^	2.155	0.634 (0.054)^a^	1.885
Private hospitals benefits (base: 50%)	70%	0.542 (0.073)^a^	1.719	0.633 (0.078)^a^	1.883	0.665 (0.072)^a^	1.944	0.489 (0.080)^a^	1.631	0.467 (0.071)^a^	1.595	0.752 (0.081)^a^	2.121
	90%	0.949 (0.075)^a^	2.583	1.146 (0.084)^a^	3.146	1.145 (0.076)^a^	3.142	0.914 (0.083)^a^	2.494	0.838 (0.075)^a^	2.312	1.300 (0.085)^a^	3.669
Outpatient benefits (base: 50%)	70%	0.311 (0.069)^a^	1.365	0.230 (0.075)^b^	1.259	0.339 (0.068)^a^	1.404	0.193 (0.075)^c^	1.213	0.231 (0.067)^a^	1.260	0.339 (0.077)^a^	1.404
	90%	0.571 (0.073)^a^	1.770	0.558 (0.077)^a^	1.747	0.674 (0.070)^a^	1.962	0.415 (0.08)^a^	1.514	0.537 (0.071)^b^	1.711	0.594 (0.079)^a^	1.811
Dental coverage (base: 40%)	70%	0.414 (0.106)^a^	1.513	0.500 (0.119)^a^	1.649	0.575 (0.107)^a^	1.777	0.289 (0.119)^c^	1.335	0.281 (0.105)^a^	1.324	0.699 (0.122)^a^	2.012
Rehabilitation and para-clinical benefits (base: 50%)	70%	0.04 (0.091)	1.041	-0.148 (0.100)	0.862	-0.058 (0.090)	0.944	-0.017 (0.102)	0.983	-0.002 (0.090)	0.998	-0.118 (0.102)	0.889
	90%	0.491 (0.081)^a^	1.634	0.480 (0.089)^a^	1.616	0.469 (0.081)^a^	1.598	0.502 (0.089)^a^	1.652	0.406 (0.081)^a^	1.501	0.571 (0.089)^a^	1.770
Long-term care benefits (base: 50%)	70%	0.244 (0.102)^c^	1.276	0.273 (0.111)^b^	1.314	0.121 (0.100)	1.129	0.474 (0.115)^a^	1.606	0.296 (0.102)^b^	1.344	0.23 (0.112)^c^	1.259
	90%	0.377 (0.105)^a^	1.458	0.348 (0.114)^b^	1.416	0.188 (0.103)	1.207	0.613 (0.117)^a^	1.846	0.389 (0.104)^a^	1.476	0.349 (0.116)^b^	1.418
Medical devices benefits (base: 60%)	90%	0.162 (0.070)^c^	1.176	0.264 (0.074)^a^	1.302	0.235 (0.067)^a^	1.265	0.169 (0.078)	1.184	0.209 (0.070)^b^	1.232	0.209 (0.075)^b^	1.232
Premium (per 10 000 IRRs)	**-**	-0.033 (0.005)^a^	0.968	-0.040 (0.005)^a^	0.961	-0.033 (0.005)^a^	0.968	-0.039 (0.005)^a^	0.962	-0.042 (0.005)^a^	0.959	-0.029 (0.005)^a^	0.971
Observations		5232		4368		5360		4240		5264		4336	
McFadden's R^2^		0.1907		0.196		0.192		0.197		0.185		0.21	
Log likelihood		-1467		-1216		-1501		-1180		-1486		-1186	
LR chi2(1^2^)		691		593		713		580		675		632	
Prob > chi2		<0.001		<0.001		<0.001		<0.001		<0.001		<0.001	

Abbreviations: SE, standard error; OR, odds ratio. ^a^ *P* < .00, ^b^*P* < .01, ^c^*P* < .05

*Preferences by age:* The cost coverage of the inpatient services resulted in the highest utility for both age groups. The coefficient of the public hospitals’ benefits for those whose age was 41 years and younger (OR = 1.98) and for those over the age of 41 years (OR = 2.10) were nearly equal, while the utility from the private hospital benefits at 70% and 90% cost coverage were higher for the younger age group.

The younger age group had higher utilities of the outpatient and dental services than their counterpart. In contrast, the older age group’s utility level of long-term care benefits was relatively higher (Table 5). At 70% cost coverage, both age groups had statistically insignificant (*P* > .05) disutility of the rehabilitation and para-clinical benefits. However, the older and younger age groups showed about an equal level of disutility of the premium (OR =  0.97 vs. 0.96). At 70% cost coverage, all the attributes except for the rehabilitation and para-clinical benefits were statistically significant (*P* < .05).

*Preferences by educational level:* The lower educated group had relatively a higher utility of the public hospitals’ benefits than the higher educated group (OR = 2.15 vs. 1.88). The utility level from all the attributes except the long-term care was higher for the higher educated group than for the lower educated one (Table 5). Both groups had higher differences in the level of utilities from the private hospitals’ benefits and dental benefits than in the other attributes. At 90% cost coverage, the higher educated had a higher utility level of the private hospital benefits than the lower educated group (OR = 3.67 vs. 2.31). Nevertheless, the more educated group’s utility level of the dental benefits was higher than the lower educated one (OR = 2.01 vs. 1.32).

*Preferences of household head:* The heads of the households had more utility from the coverage of the costs. For example, at 90% cost coverage the households’ heads had a higher utility of the public hospitals’ benefits than the non-head households’ utility (OR = 2.20 vs. 1.87), while the heads of the households had a lower utility of the private hospitals’ benefits than the non-head households (OR = 2.40 vs. 3.34).

At 70% cost coverage for the dental services, the non-heads category of households had a higher utility than the household heads category (OR = 1.71 vs. 1.45), while both the head and non-head household groups had disutility from the rehabilitation and para-clinical services (OR = 0.98 vs. 0.93). The disutilities from the premium for both groups (OR = 0.96 vs. 0.97) were almost equal (Table 6).

**Table 6:** Health Insurance Attributes Preferences of Participants by Status in Household, Income, and Health in Tehran, Iran; 2016

**Attributes**	**Cost Coverage**	**Status in Household**				**Household Income (Monthly)**						**Health Status**			
		**Head of Household**		**Non-head Household**		**< 20 Million Rials**		**20-40 Million Rials**		**> 40 Million Rials**		**Have a Chronic Condition(s)**		**Healthy**	
		**β (SE)**	**OR**	**β (SE)**	**OR**	**β (SE)**	**OR**	**β (SE)**	**OR**	**β (SE)**	**OR**	**β (SE)**	**OR**	**β (SE)**	**OR**
Public hospitals benefits (base: 60%)	90%	0.786 (0.051)^a^	2.195	0.627 (0.051)^a^	1.872	0.816 (0.052)^a^	2.261	0.628 (0.064)^a^	1.874	0.549 (0.085)^a^	1.732	0.749 (0.049)^a^	2.115	0.653 (0.053)^a^	1.921
Private hospitals benefits (base: 50%)	70%	0.487 (0.075)^a^	1.627	0.685 (0.076)^a^	1.984	0.512 (0.076)^a^	1.669	0.643 (0.093)^a^	1.902	0.764 (0.132)^a^	2.147	0.522 (0.071)^a^	1.685	0.67 (0.081)^a^	1.954
	90%	0.875 (0.078)^a^	2.399	1.207 (0.081)^a^	3.343	0.905 (0.080)^a^	2.472	1.145 (0.099)^c^	3.142	1.307 (0.133)^a^	3.695	0.984 (0.075)^a^	2.675	1.11 (0.084)^a^	3.034
Outpatient benefits (base: 50%)	70%	0.344 (0.071)^a^	1.411	0.200 (0.072)^b^	1.221	0.192 (0.071)^b^	1.212	0.35 (0.090)^a^	1.419	0.401 (0.124)^a^	1.493	0.259 (0.067)^a^	1.296	0.291 (0.076)^a^	1.338
	90%	0.552 (0.076)^a^	1.737	0.571 (0.073)^a^	1.770	0.606 (0.074)^a^	1.833	0.563 (0.093)^a^	1.756	0.46 (0.129)^a^	1.584	0.540 (0.072)^a^	1.716	0.596 (0.077)^a^	1.815
Dental coverage benefits (base: 40%)	70%	0.368 (0.111)^b^	1.445	0.539 (0.113)^a^	1.714	0.388 (0.110)^a^	1.474	0.399 (0.142)^b^	1.490	0.781 (0.201)^a^	2.184	0.463 (0.105)^a^	1.589	0.438 (0.12)^a^	1.550
Rehabilitation and para-clinical benefits (base: 50%)	70%	-0.017 (0.095)	0.983	-0.077 (0.096)	0.926	-0.039 (0.095)	0.962	-0.070 (0.119)	0.932	-0.039 (0.166)	0.962	-0.062 (0.091)	0.940	-0.028 (0.1)	0.972
	90%	0.474 (0.084)^a^	1.606	0.491 (0.085)^a^	1.634	0.458 (0.085)^a^	1.581	0.512 (0.106)^a^	1.669	0.538 (0.14)^b^	1.713	0.458 (0.081)^a^	1.581	0.521 (0.088)^a^	1.684
Long-term care benefits (base: 50%)	70%	0.353 (0.107)^b^	1.423	0.178 (0.106)^b^	1.195	0.226 (0.106)^a^	1.254	0.177 (0.136)	1.194	0.538 (0.176)	1.713	0.378 (0.102)^a^	1.459	0.110 (0.111)	1.116
	90%	0.446 (0.109)^a^	1.562	0.292 (0.109)^b^	1.339	0.282 (0.109)^c^	1.326	0.361 (0.135)^b^	1.435	0.631 (0.191)^b^	1.879	0.489 (0.104)^a^	1.631	0.190 (0.114)	1.209
Medical devices benefits (base: 60%)	90%	0.193 (0.073)^b^	1.213	0.222 (0.071)^b^	1.249	0.245 (0.073)^a^	1.278	0.220 (0.089)^c^	1.246	0.121 (0.122)^b^	1.129	0.262 (0.07)^a^	1.300	0.136 (0.074)	1.146
Premium (per 10 000 IRRs)	**-**	-0.038 (0.005)^a^	0.963	-0.034 (0.005)^a^	0.967	-0.041 (0.005)^a^	0.960	-0.037 (0.006)^a^	0.964	-0.021 (0.008)^a^	0.979	-0.039 (0.005)^a^	0.962	-0.031 (0.005)^a^	0.969
Observations		193		0.195		4896		3024		1680		5408		4192	
McFadden's R^2^		-1342		-1338		0.199		0.195		0.198		0.195		0.18	
Log likelihood		643		649		-1358		-843		-466		-1496		-1191	
LR chi2(1^2^)		<0.001		<0.001		679		409		231		755		522	
Prob > chi2		2785		2779		<0.001		<0.001		<0.001		<0.001		<0.001	

Abbreviations: SE, standard error; OR, odds ratio. ^a^ *P* < .00, ^b^*P* < .01, ^c^*P* < .05

*Preferences by income:* The findings indicated a positive association between the utility of most of the attributes and the participants’ monthly income. At 90% cost coverage, those with a monthly income of more than 40 million IRRs had the highest utility of the private hospitals’ benefits (OR = 3.69) than those in the other income categories. In contrast, while those with a monthly income of fewer or equal to 20 million IRRs had the highest utility of the public hospitals’ benefits (OR = 2.26). Again, those with a monthly income of fewer or equal to 20 million IRRs showed an increased likelihood of utility of the outpatient services from OR = 1.21 to OR = 1.83 with an increase in cost coverage from 70% to 90%.

The disutilities from the para-clinical and rehabilitation services and the premium was almost equal for all the income groups. That is, the premium showed less sensitivity to the income of the individuals. The utilities of the participants in the low and middle-income groups at 70% cost coverage for the para-clinical and rehabilitation and the high-income people with the long-term care at the same level of cost coverage were statistically insignificant (*P* > .05).

*Preferences by health status:* Both the healthy individuals and individuals with chronic diseases had higher utilities from public and private hospitals benefits (inpatient services) than the utilities obtained from the other attributes. The probability of utilities from the public inpatient services among the patient group (OR = 2.12) and healthy group (OR = 1.92) showed slight difference. However, the healthy group had a higher utility from the private inpatient services at 90% cost coverage than the patient group (OR = 3.03 vs. 2.67). Both the healthy and patient groups had almost equal utility from the outpatient services (OR = 1.82 vs. 1.72), and rehabilitation and para-clinical services (OR = 1.68 vs. 1.58), while the patient group had a higher utility from long-term care than the healthy group (OR = 1.63 vs. 1.21).

## Discussion

This study elicited the insured Iranians’ preferences about the different attributes of health insurance plans in Iran. Our findings revealed an increase in utility related to the increase in cost coverage for all the attributes analyzed, and the highest utility from the private hospitals’ benefits followed by the public hospitals’ benefits, and outpatient benefits, respectively. The quality of private healthcare services in Iran is assumed to be better than public healthcare services.^25^ The utility of cost coverage of private inpatient services was higher than the public hospitals’ services. However, the households’ heads had a higher utility from the public hospital benefits. Others reported a higher utility of the less educated from the public hospital benefits and disutility of the higher premium.^26^ Also, the high expenditure of the households’ heads was one of the main reasons for the lower utility from private hospital benefits.^27^ The utilization of private hospital services was also associated with a higher probability of incurring catastrophic health expenditures (CHEs).^28^

Others reported the likely more preferences to both the public and private providers than to one of the providers alone.^8^ The individuals’ higher preferences to the private than to the public hospitals’ benefits at the 90% cost coverage observed in our study may imply better quality services of the private providers. Also, the utility from the use of private services was higher among high-income households, and the high hospital admission rate in Iran might reflect the higher socioeconomic status of those individuals with the admission need. That is, people with higher socioeconomic status are likely to have better access to healthcare in general, and inpatient services in particular. The difference in health insurance coverage might be one of the main reasons for such variations.^29^ Thus, improving health insurance coverage increases the utilization of healthcare services.^29-31^ A study in Ethiopia also revealed peoples’ preference for health insurance packages with access to both the public and private providers.^32^

Gender of the insured participants was one of the main factors which affected preference. At the highest level of cost coverage, the males’ preference for public hospitals’ benefits was considerably higher than that of the females,’ while at the same rate of cost coverage, females had a markedly higher preference for the private hospitals’ benefits than the males do. Nevertheless, the disutility of both genders from the premium was almost the same. Evidence revealed a higher preference of women for outpatient services and men for inpatient services. Like in ours, the disutility from the premium was the same for both men and women.^26^

The findings also revealed an age difference in utilities of the health insurance benefits. The younger individuals’ utilities from the coverage of the private hospitals’ benefits, outpatient and dental coverage benefits were higher than the utilities of the older ones. The impact of age on household choice for insurance is found to be non-linear and U-shaped.^33^ The older individuals in our findings revealed a higher utility from the long-term benefits than their counterpart, which might be due to the occurrence of aging-related chronic diseases.^34^ In Japan’s public sector, the provision of mandatory long-term care insurance was in place since 2000 to ensure better accessibility of healthcare services to the elderly and reduce the healthcare-related burden on the families of the elderly. However, its sustainability was challenging because of financial constraints.^35^ The premium of the available small private market for long-term health insurance was also expensive compared to its benefits.^36^ Again, those who have more coverage were likely to frequently utilize more medical services than those with less coverage.^37^ Thus, income is one of the main factors which determine health insurance preference of health expenditures of people.^38^ Our findings indicated the odds of choosing health insurance with more cost coverage among the higher income groups, which is consistent with the findings which reported wealthier individuals are more likely to seek and prefer for the private than public medical services.^39^

The findings indicated a higher utility from the dental services with higher cost coverage and the more preference for the dental care benefits insurance coverage among the more educated individuals. Similarly, a study in Namibia reported an association between educational status and health insurance,^40^ and an association between more dental health-care services utilization and several factors including aging, having dental insurance, higher income, being a university graduate, self-rated poor oral health and not regularly brushing own teeth.^41^ In Iran, there are different sources for the insurance of the health benefits packages.^42^ Thus, the high complementary health insurance coverage (>35%) observed in our findings might inform the uniform provision of social insurance benefits and the lack of diversity of the benefits packages to the citizens. This condition might have resulted from the influence of the individuals’ decisions in purchasing the health insurance benefit packages by several factors including the demographic, socioeconomic and past health services utilization.^14^

The low utility from the rehabilitation and para-clinical services revealed in our findings might be related to the limited choices and low quality and quantity of the services available.^43^ These conditions, in turn, might have influenced the individuals’ preferences, and the cost coverage of the services. Other DCE related studies in high and low-income countries reported that the benefits of the services and the extent of the cost coverage had marked influence on the individuals’ choices.^27,44,45^ The uncertainty of whether to utilize the healthcare services or not and the CHEs related to the utilization of the services can also influence the individuals’ rationale decision in selecting the benefit packages.^44^ Our findings showed that the individuals’ desire for full coverage of the health benefits package even to the services not included in the insurance benefit packages, and equal premium to all approach is a known source of dissatisfaction.^46^

Despite the insignificant relationship between the participants with chronic health conditions and their preferences to most of the attributes analyzed, there was a significantly higher preference for the long-term care benefit packages. Besides, those participants with chronic conditions were older than healthy individuals. The chronic health conditions are among the main factors which affect preferences.^47,48^ Individual’s health status and purchasing of health insurance are inversely related and can, in turn, affect the health benefits packages utilization. Also, healthy individuals usually purchase more private health benefits packages insurances than public ones. Again, evidence shows that despite the complementary and voluntary benefits packages, health insurance purchasing may not be affected by an individual’s health risks.^49^ In contrast, risk-averse individuals seek to get utility by purchasing health insurance to avoid the consequences of the risks.^50^ A person with poor health condition, especially among the self-employed, is more likely to experience financial problems and challenges to purchase health insurance.^51^ Thus, securing insurance for the health benefits packages from the public sector can help protect the poor against CHEs and improve health outcomes.^52^

This study attempted to investigate the health insurance preferences of insured people in Iran. The findings provided useful information for health policy-makers and HIOs to better understand and improve the design of the existing health insurance plans in the context. However, the analysis focused on conveniently obtained data of a limited geographic area. Thus, our findings necessitate cautious interpretations. With this assumption that previous evidence and experts can reflect the opinions of insured people about attributes of health insurance, we excluded them from the first phase of the study.

## Conclusion

The findings revealed that the participants tended to choose health insurance plans with higher cost coverage for healthcare services and with lower premiums. However, the individuals’ health insurance preferences were subject to the influences of the demographic characteristics, income, and health status. The findings are believed to contribute to the designing of better health insurance policies in the context of Iran, improve the participation of the citizens in health insurance, and increase the utility of the insured from the insurance benefits packages.

## Acknowledgments

The authors would like to thank Prof. Arash Rashidian for his scientific suggestions in developing and designing this study. The authors would also like to thank Tehran University of Medical Sciences, Tehran, Iran (Fund No. 29725), and Social Security Research Institute, Tehran, Iran (Fund No. 395002684) for their financial support, and the SSIO and Iran HIO for their relentless cooperation in providing us with the needed data for the analysis.

## Ethical issues

This study was approved by Ethics Committee for Tehran University of Medical Sciences, Tehran, Iran (9121504005).

## Competing interests

Authors declare that they have no competing interests.

## Authors’ contributions

AKK and AAS were involved to designing the study. AKK was responsible for cleaning data and running statistical analysis and interpretation of the results. AKK and AW were responsible for writing the first draft. All authors contributed equally to editing draft versions and accept full responsibility for the content of the manuscript.

## Authors’ affiliations

^1^Research Center for Environmental Determinants of Health, Health Institute, Kermanshah University of Medical Sciences, Kermanshah, Iran. ^2^Department of Health Economics and Management, School of Public Health, Tehran University of Medical Sciences, Tehran, Iran. ^3^School of Public Health, College of Health Sciences, Mekelle University, Tigray, Ethiopia.

## Key messages

**Implications for policy makers**

- Generally, the participants are willing to choose health insurance plans with higher cost coverage of healthcare services and with lower premiums.
- The private hospitals’ benefits followed by the public hospitals’ benefits provided the highest utility for all sub-categories of the insured respondents than the other attributes.
- The cost coverage of the dental services, long-term care, and para-clinical and rehabilitation services provided the lower utilities for the participants.

**Implications for public**

Evidence concerning health insurance preferences of the insured people is useful for improving and designing better benefits packages. Health insurance with a higher cost coverage of the public and private hospitals’ (inpatient) and outpatient services benefits can provide more utility to people. The service users might consider the insurance for the health benefits packages such as dental care as less important. Thus, providing them with the opportunity for more options for basic benefits packages could have increased their utilities.
